# Observation and assessment of the immediate use of a silicon hydrogel contact lens after transepithelial corneal cross linking: a prospective study

**DOI:** 10.1186/s12886-023-03217-4

**Published:** 2023-11-20

**Authors:** Liu Liu, Teruko Fukuyama, Tian Han, Zhe Zhang, Lin Jiang, Yanlan Ding, Xingtao Zhou, Jifang Wang

**Affiliations:** 1 Department of Nursing, Eye & ENT Hospital, Fudan University, 83 Fenyang Road, Shanghai, 200031 China; 2 Department of Ophthalmology and Optometry, Eye & ENT Hospital, Fudan University, 83 Fenyang Road, Shanghai, 200031 China; 3 Shanghai Research Center of Ophthalmology and Optometry, 83 Fenyang Road, Shanghai, 200031 China

**Keywords:** Transepithelial corneal cross liking, Corneal contact lens, Comfortability, Ophthalmologic care

## Abstract

**Background:**

Transepithelial corneal crosslinking (CXL) is a novel surgical approach for the treatment of keratoconus, which is a bilateral asymmetrical ophthalmological disease accompanied by progressive corneal ectasia. Silicon hydrogel (SiH) contact lenses have been extensively used in clinical ophthalmologic medicine, as a postoperative ophthalmological intervention. However, the ideal lens application duration after transepithelial CXL remains uncertain. Here, we aimed to investigate the effects and comfort of immediate corneal contact lens use after transepithelial CXL for keratoconus.

**Methods:**

In this prospective study, 60 patients with keratoconus who underwent transepithelial CXL treatment were enrolled from September 2021 to January 2023 with a male:female ratio of 39:21, and an average age of 25.42 ± 5.47 years. The patients were divided randomly into two groups: group A contained 30 patients wearing silicone hydrogel contact lenses for 7 days postoperatively, and group B contained 30 patients wearing the same contact lenses for 3 days.

Ten subjective ophthalmologic symptoms were surveyed by the patients, including pain, photophobia, foreign body sensation, tearing, burning, blurred vision, dry eyes, difficulty opening the eyes, astringency, and stinging. Ophthalmologic signs, including corneal edema and conjunctival congestion, were recorded by a single clinician on postoperative days 1, 3, and 7.

**Results:**

Each surgical procedure was readily performed without complications, and both groups postoperative day 7 (*P* = 0.04), where group B scored (0.01 ± 0.41) lesser than group A (0.12 ± 0.29), whilst corneal edema in both groups recorded significantly different on postoperative days 5 and 7 (group A demonstrated the result of 0.17 ± 0.14 and 0.08 ± 0.11 for the respective days, whereas group B indicated 0.10 ± 0.13 and 0.03 ± 0.07 at the corresponding times).

**Conclusions:**

Immediate use of silicone hydrogel corneal lenses after transepithelial CXL effectively alleviates postoperative ocular distress, particularly with a three-day use period as the ideal duration.

## Background

Keratoconus is a bilateral asymmetrical ophthalmological disease accompanied by progressive corneal ectasia. The major clinical characteristics are substantial thinning and bulging of the cornea, as well as astigmatism to various extents, resulting in a rapid reduction in visual acuity.

The prevalence and incidence rates are approximately 1:375, and are distributed globally [1]. There is no bias for disease occurrence in both sexes and it affects varying ethnicities to different degrees  [2, 3].

Corneal crosslinking (CXL) is regarded as an effective clinical treatment for preventing disease progression [4]. Transepithelial CXL is a novel surgical approach for the treatment of keratoconus. Unlike traditional CXL, transepithelial CXL does not involve removal of the corneal epithelium [5].

Although the corneal epithelial layer is untouched during transepithelial CXL procedures, topical destruction caused by riboflavin infiltration and UV light exposure may lead to postoperative pain, photophobia, and foreign body sensation in patients [6]. Therefore, it is essential to provide postoperative ophthalmological interventions such as wearing corneal contact lenses [7].

The main component of the silicon hydrogel (SiH) corneal contact lens is silicone hydrogel, containing 38% hydrogen, a diameter of 14 mm, with base curve 8.8 mm, and corneal central thickness of 0.07 mm [8–10]. Compared with other contact lenses, this particular lens is smooth with high oxygen permeability, which effectively facilitates the repair of the corneal epithelial layer, providing satisfactory postoperative results for patients [8–11].

The US Food and Drug Administration (FDA) has approved the overnight use or continuous wearing of contact lenses as part of the treatment of keratoconus [12]. Therefore, SiH contact lenses have been extensively used in clinical ophthalmologic medicine. However, the ideal lens application duration after transepithelial CXL remains uncertain.

Therefore, in this study, we investigated the impact of contact lens application after transepithelial CXL to analyze whether the patient’s overall comfort is related to the duration of lens wearing and to further evaluate patient satisfaction based on the attained ophthalmologic evidence.

## Methods

### Participants

Patients received transepithelial CXL between September 2021 and January 2023 and were selected from the Fudan University-affiliated Eye and ENT hospital. The inclusion criterion was clear diagnosis of spontaneous keratoconus, pediatric keratoconus, increase of maximum keratometry by at least 1 Diopter (D) or increase of pachymetry by at least 20 microns in a year,

preoperative topography (examined with a Pentacam produced by Oculus Optikgeräte, Wetzlar, Germany) results indication of the thinnest corneal thickness > 380 um, and age ranges between 18–35 years old. Carriers of systemic diseases, autoimmune diseases, or psychological diseases were excluded. The evaluation of lens comfort was conducted by an experienced ophthalmologist, observed via a slit-lamp, and postoperative corneal morphologies were recorded for each individual.

In this prospective study, 60 participants (60 eyes) were recruited, with an average age of 25.42 ± 5.47 years. The male:female ratio was 39:2. The patients were divided into groups A and B based on the random number table method, and each group contained 30 patients. There were no significant differences between the two groups (*P* > 0.05) as indicated in Table 1.

**Table 1:** Participants’ basic information for two groups (Mean ± SD, *N* = 60)

	7 days post-surgery	3 days post-surgery	*P* value
Age	25.50 ± 5.05	25.33 ± 5.90	0.91
Sex (male/female)	19/11	20/10	1.00
Kmax (D)	59.34 ± 8.15	59.80 ± 9.66	0.84
Corneal thinnest point	443.8 ± 41.39	449.0 ± 37.45	0.61

*Kmax* Maximum keratometry

### Study protocol

After a routine preoperative examination, each surgery was performed by the same experienced senior physician.

The surgical procedure was the same as that used in our previous study [13]. The cornea was infiltrated with Sufficient Paracel (Avedro, Waltham, MA, USA) which includes 0.25% riboflavin-5-phosphate, NaCl, 1.2% hydroxypropyl methylcellulose (HPMC), 0.01% benzalkonium chloride, sodium edetate and trometamol for 14 min, then exposed to UV light with a wavelength of 370 nm and intensity of 45 mW/cm2 (Avedro, Waltham, MA, USA). The exposure time was 5 min 20 s for pulse irradiation with 1 s bright and 1 s dark, and the total energy was 7.2 J/cm2.

Once the surgical procedure was completed, both groups were immediately fixed with AcuVue Oasys SiH contact lenses (Johnson&Johnson, ACUVUE, USA). In terms of postoperative medication use, each patient was provided with Clopito (Santen, Japan) and Flumei drops (Santen, Japan) after surgery, accompanied with a follow up period of 7 days.

On the day after the operation, the researcher evaluated the lens condition of each patient using a slit-lamp microscope, including the central position of the lens, any horizontal or vertical lens displacement, degree of lens movement during natural blinking in the original ocular position and upward gaze position, and wettability of the lens. Patients in Group A underwent lens removal at postoperative day 7, whereas those in Group B underwent lens removal after 3 days.

### Postoperative observation

#### Subjective symptoms of the operative eye

##### Postoperative comfortability

A standard self-administered questionnaire was adopted for both groups to record the postoperative comfort of the ocular area, which was administered on postoperative days 1, 3, 5, and 7. The questionnaire assessed 10 symptoms, including photophobia, tearing, burning, pain, foreign body sensation, blurred vision, difficulty opening the eyes, dry eyes, edema, and sting. Each feature was scored from 0–3, where a score of 0 was considered asymptomatic, and scores of 1, 2, and 3 were rated as mild, moderate, and severe, respectively. The participants received a tutorial regarding the questionnaire, and the completed questionnaires were collected on the day of the re-examination.

##### Severity of postoperative ocular pain

The visual analogue scale (VAS) is used to assess the severity of postoperative ocular pain. The VAS for pain is composed of a 10 cm straight line, which is marked with 0 at one end and 10 at the other. A score of 0 represents “no pain,” score 1–3 represents “mild pain,” which does not affect sleeping; score 4–6 is defined as “moderate pain,” which affects night sleep; score 7–10 is regarded as “severe pain,” which severely affects sleeping [14]. The participants were asked to rate their scores based on their subjective pain sensation.

#### Objective signs of the operative eye

1. A single blinded test was applied for corneal edema assessment, where one senior ophthalmologist masked the allocation of the participant’s group while examining the ocular condition of each participant using slit-lamp microscopy. The resulting ocular status was recorded using a score range between 0–3. A higher score represents worse symptoms, where 0 implies a symptom-free cornea and 3 indicates the most edematous cornea. In addition, conjunctival congestion was examined by the same experienced doctor via slit-lamp using the same single blinding method and the ophthalmologic status was recorded. Scoring ranged 0–3, and the higher the score, the worse the conjunctival congestion.
2. Corneal contact lens conditions were also recorded at each examination.

### Statistical analysis

The database was established in Excel using SPSS 20.0 statistical software. All the data were tested for homogeneity of variance and sphericity. Further, analysis of variance (ANOVA) and chi-square tests were used for comparison of preoperative basic condition in the two groups, and repeated measures analysis of variance (rANOVA) and one-way ANOVA were used for postoperative comparison between groups; *P* < 0.05 was considered statistically significant. Lens displacements from the center were analyzed using the mean standard deviation.

## Results

All surgical procedures were performed smoothly without any complications. In total, 60 patients in groups A and B completed the entire period of lens wearing and observation.

### Adaptability of the Acuvue Oasys contact lens

The center of the lens was well accommodated as it evenly covered the entire cornea. The degree of horizontal and vertical displacement of the lens center was within 1 mm, and the necessary mobility for lens in situ and upward gazing positions was sufficient, which were 0.61 ± 0.38 mm and 0.88 ± 0.12 mm, respectively. No accidental lens detachment occurred, and the wetness of the lens was ample.

### Patient ocular subjective symptom score

#### Subjective signs of both groups

**Fig. 1:**
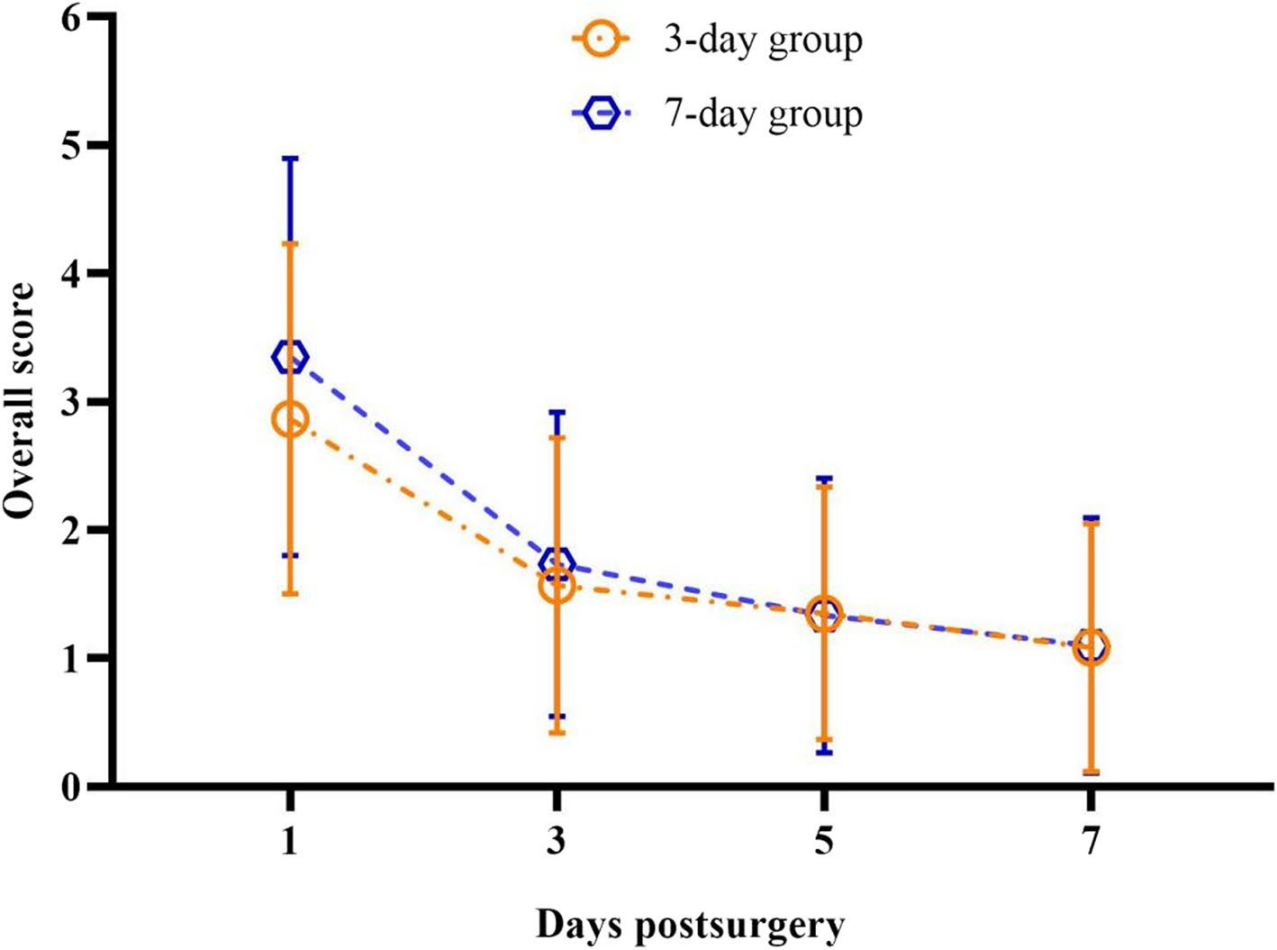
Comparison of the total score of postoperative subjective symptoms between the two groups

The subjective signs for both groups were thoroughly examined with the corresponding time effect, indicating obvious statistical differences between the two groups (*P* < 0.001), whereas the grouping effect was not statistically significant (*P* = 0.50), as illustrated in Table 2 and Fig. 1.

**Table 2:** Comparison of the total score of subjective symptoms between the two groups (Mean ± sd, *N* = 60)

Group	n	Post-op				Time effect F	Group effect F	Group *time F
		1d	3d	5d	7d			
A (7)	30	3.35 ± 1.55	1.73 ± 1.19^a^	1.33 ± 1.07^ab^	1.10 ± 0.99^ab^			
B (3)	30	2.87 ± 1.36	1.57 ± 1.15^a^	1.35 ± 0.98^a^	1.08 ± 0.97^a^	43.58	0.49	0.35
*P*		0.205	0.583	0.950	0.948	< 0.001	0.50	1.12

Group A wearing the lens for 7 days whilst group B for 3 days

^a^vs Post-op 1d, *P* < 0.05

^b^vs Post-op 3d, *P* < 0.05

The total subjective symptom scores in the two groups demonstrated a downward trend with time, stipulating the highest score on postoperative day 1, followed by a significant reduction on postoperative day 3; only a few decreases were observed from postoperative days 3 to 5 and 5 to 7. The distinguishing symptoms that patients reported postoperatively were "foreign body sensation,” “dry eyes,” “pain,” and "photophobia.” In particular, 88.3% of participants complained of foreign body sensation, 72% had dry eyes, 61% had photophobia, and 42% patients with varying degrees of pain.

Further, at postoperative day 7, score of “pain” indicated an obvious statistical difference between two groups (*P* = 0.04), where the score of group B (0.01 ± 0.41) with lens removal after postoperative day 3 was distinctly lesser in pain than that of group A (0.12 ± 0.29) with lens removal after postoperative day 7. The corneal edema score for the two groups manifested a statistical difference on postoperative days 5 and 7 (*P* = 0.04, *P* = 0.02), where group B scored lower than group A.

#### Scores of each postoperative subjective symptoms of the two groups

**Fig. 2:**
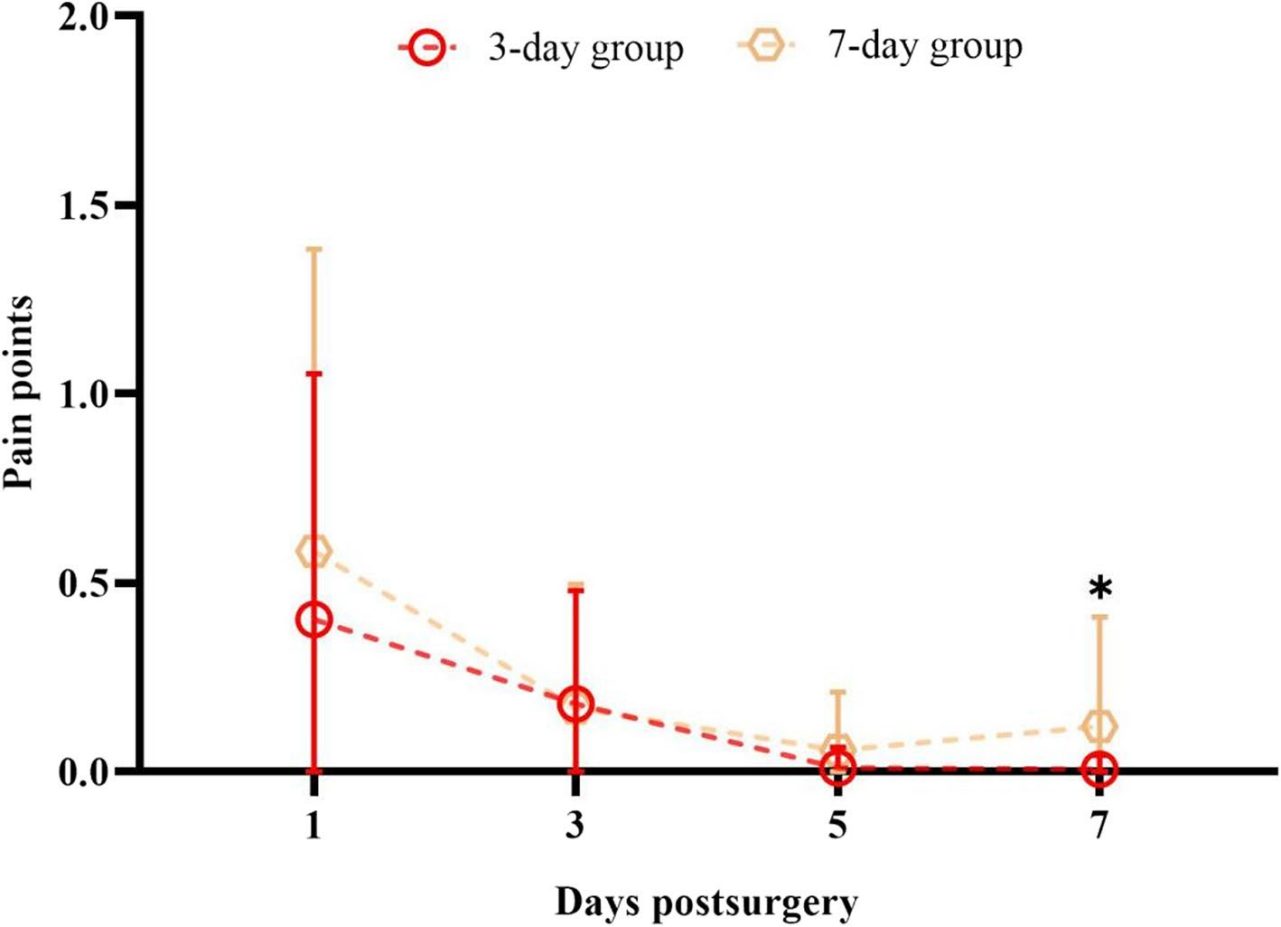
Comparison of pain scores between the two groups

There was no significant grouping effect on the total subjective symptom scores between the two groups (*P* > 0.05) (Table 3). However, the analyzed data indicated a statistical difference in the "pain" score of the two groups on postoperative day 7 (*P* = 0.04), where patients in group B experienced less pain (0.01 ± 0.41) than did in group A (0.12 ± 0.29) (Table 4, Fig. 2).

**Table 3:** Score of subjective symptoms of both groups after trans CXL procedure (mean ± sd, *N* = 60)

	Time after	Group		*P*	Time effect		Group * time effect	
	Surgery	A(7)	B(3)		F	*P*	F	*P*
Photophobia	1d	0.50 ± 0.47	0.60 ± 0.49	0.50	-		-	
	3d	0.18 ± 0.36	0.21 ± 0.38	0.78	-		-	
	5d	0.13 ± 0.32	0.15 ± 0.34	0.82	-		-	
	7d	0.08 ± 0.23	0.02 ± 0.10	0.30	22.56	< 0.001	0.92	0.43
Tearing	1d	0.35 ± 0.46	0.27 ± 0.41	0.55	-		-	
	3d	0.13 ± 0.32	0.10 ± 0.28	0.65	-		-	
	5d	0.12 ± 0.31	0.10 ± 0.28	0.80	-		-	
	7d	0.13 ± 0.32	0.06 ± 0.22	0.51	4.28	0.009	1.37	0.26
Foreign body	1d	1.00 ± 0.37	0.81 ± 0.47	0.24	-		-	
sensation	3d	0.63 ± 0.39	0.46 ± 0.42	0.12	-		-	
	5d	0.33 ± 0.38	0.25 ± 0.32	0.38	-		-	
	7d	0.27 ± 0.37	0.35 ± 0.37	0.59	35.23	< 0.001	1.65	0.19
Burning	1d	0.10 ± 0.28	0.14 ± 0.31	0.61	-		-	
	3d	0.00 ± 0.00	0.06 ± 0.22	0.15	-		-	
	5d	0.00 ± 0.00	0.04 ± 0.14	0.13	-		-	
	7d	0.00 ± 0.00	0.00 ± 0.00	0.99	5.39	0.003	1.89	0.14
Sting	1d	0.12 ± 0.31	0.04 ± 0.20	0.33	-		-	
	3d	0.05 ± 0.20	0.04 ± 0.20	0.85	-		-	
	5d	0.05 ± 0.20	0.06 ± 0.22	0.89	-		-	
	7d	0.05 ± 0.20	0.04 ± 0.20	0.99	0.70	0.42	1.01	0.33
Astringent	1d	0.24 ± 0.44	0.16 ± 0.37	0.53	-		-	
	3d	0.09 ± 0.27	0.10 ± 0.29	0.56	-		-	
	5d	0.07 ± 0.26	0.08 ± 0.28	0.71	-		-	
	7d	0.03 ± 0.19	0.04 ± 0.20	0.99	4.42	0.008	0.25	0.86
Difficulty	1d	0.17 ± 0.38	0.21 ± 0.40	0.87	-		-	
opening eyes	3d	0.07 ± 0.25	0.08 ± 0.27	0.88	-		-	
	5d	0.07 ± 0.25	0.08 ± 0.27	0.88	-		-	
	7d	0.03 ± 0.18	0.04 ± 0.20	0.99	4.33	0.02	0.07	0.94
Edema	1d	0.19 ± 0.39	0.08 ± 0.27	0.17	-		-	
	3d	0.03 ± 0.19	0.06 ± 0.22	0.65	-		-	
	5d	0.03 ± 0.19	0.04 ± 0.20	0.92	-		-	
	7d	0.03 ± 0.19	0.04 ± 0.20	0.98	2.63	0.08	1.27	0.29
Dry eyes	1d	0.70 ± 0.43	0.56 ± 0.43	0.14	-		-	
	3d	0.55 ± 0.48	0.44 ± 0.45	0.39	-		-	
	5d	0.53 ± 0.45	0.55 ± 0.43	0.84	-		-	
	7d	0.47 ± 0.45	0.52 ± 0.44	0.77	1.40	0.26	1.75	0.17

**Table 4:** Comparison of pain scores between the two groups (Mean ± sd, *N* = 60)

Time after Surgery	Group		*P*		Time effect	Group effect	Group * time effect
	A(7)	B(3)					
Post-op 1d	0.60 ± 0.81	0.35 ± 0.60	0.34		-	-	-
Post-op 3d	0.17 ± 0.33	0.15 ± 0.26	0.82		-	-	-
Post-op 5d	0.06 ± 0.16^ab^	0.04 ± 0.14^a^	0.64	F	10.06	2.10	1.88
Post-op 7d	0.12 ± 0.29^abc^	0.01 ± 0.40^a^	0.04	*P*	< 0.001	0.15	0.15

Group A wearing the lens for 7 days whilst group B for 3 days

^a^vs Post-op 1d, *P* < 0.05

^b^vs Post-op 3d, *P* < 0.05

^c^vs Post-op 5d, *P* < 0.05

In the overall evaluation of 10 subjective symptoms (rANOVA) between the two groups A and B, except for "sting" (*P* = 0.42) and "dry eyes" (*P* = 0.26), the time effects of the score of the remaining eight symptoms showed significant statistical difference (*P* < 0.05), and the tendency declined over time; the scores of all 10 symptoms had an obvious decrease at postoperative day 3. The postoperative "dry eye" symptoms lasted the longest, and the pain of varying degrees remained high, up to 62%, for both groups on postoperative day 7.

### Ocular sign assessment under slit-lamp microscopy

**Fig. 3:**
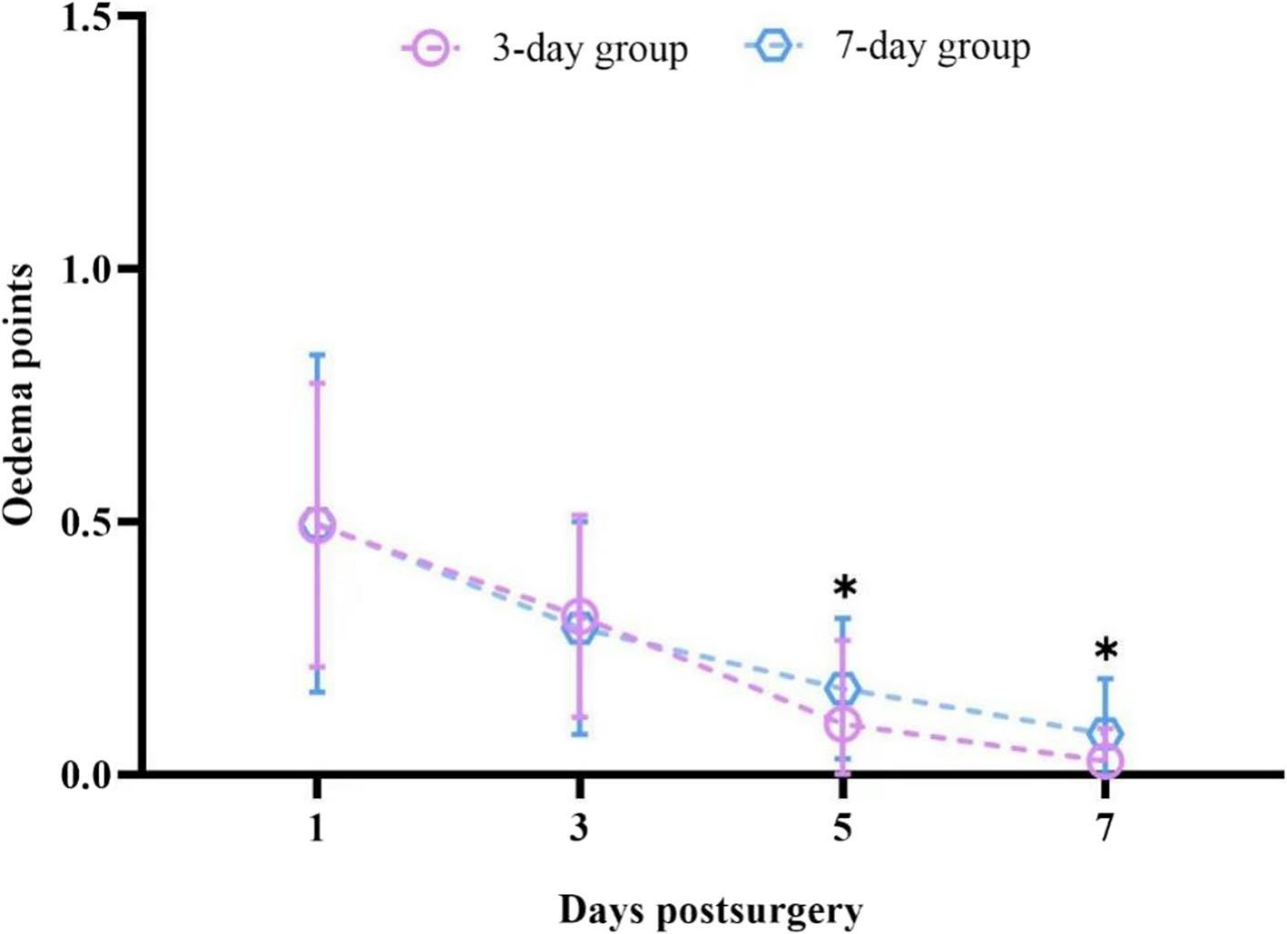
Comparison of Corneal edema score between the two groups

1. The time effect of this study demonstrated a significant statistical difference between the scores of corneal edema and conjunctival hyperemia on postoperative days 1, 3, 5, and 7 (*P* < 0.001), and its tendency decreased with time, whereas the grouping effect of ocular sign scores in the two groups was not statistically significant.
2. The corneal edema scores for both groups were most statistically significant on days 5 and 7, with *P* values of 0.04 and 0.02 correspondingly. In particular, the score of group B showed an apparent reduction compared to that of group A on these two days (Table 5, Fig. 3).

**Table 5:** Comparison of ocular signs scores under slit-lamp between the two groups (Mean ± sd, N = 60)

	Post-op	Group		*P*		Time effect	Group effect	Group*time effect
	duration	A (7)	B (3)					
Corneal edema	1d	0.50 ± 0.33	0.48 ± 0.28	0.97				
	3d	0.29 ± 0.21	0.29 ± 0.20^a^	0.80				
	5d	0.17 ± 0.14^ab^	0.10 ± 0.13^a^	0.04	*P*	48.96	0.16	0.56
	7d	0.08 ± 0.11^abc^	0.03 ± 0.07^abc^	0.02	F	< 0.001	0.68	0.65
Conjunctival	1d	0.36 ± 0.34	0.35 ± 0.19	0.89				
congestion	3d	0.18 ± 0.15^a^	0.17 ± 0.14^a^	0.71				
	5d	0.13 ± 0.12^a^	0.10 ± 0.12^ab^	0.35	*P*	20.13	0.27	0.07
	7d	0.08 ± 0.13^ab^	0.05 ± 0.09^ab^	0.23	F	< 0.001	0.60	0.97

Group A wearing the lens for 7 days whilst group B for 3 days

^a^vs Post-op 1d, *P* < 0.05

^b^vs Post-op 3d, *P* < 0.05

^c^vs Post-op 5d, *P* < 0.05

## Discussion

Ensuring patient comfort and satisfaction is a top priority in healthcare systems. Application of bandage contact lens could effectively promote epithelialization and ease the postoperative symptoms after corneal crosslinking [15–18]. Though there is postoperative care solely applying pharmacological agents such as topical NSAIDS, corticosteroids, and anesthetics [19]. Yet, to the best of our knowledge, exclusive use of pharmacological agents postoperatively could not shorten the time of recovery or relieve the postoperative symptoms. In this study, we explored the possibility of shortening the postoperative wear time of contact lenses using highly oxygen-permeable lenses after transepithelial CXL surgery.

Through rigorous observation, we discovered that it was feasible for patients to remove their lenses only 3 days after the procedure. This finding has the potential to significantly reduce the interval between follow-up visits and increase patient satisfaction, particularly for those traveling from other provinces or suburban areas to receive medical treatment.

In subjective ocular assessment, patients in group B, who removed the lens after 3 days, experienced less ocular pain at day 7 with a score of 0.01 ± 0.40 than those in group A, who removed the lens after 7 days with score of 0.12 ± 0.29. As pain is one of the concerning postoperative matters in CXL surgery, the results of the present study could serve as a reliable reference for future keratoconus post-CXL care. In terms of subjective ocular signs, group B displayed lower scoring results than group A in postoperative corneal edema for day 5 and 7, which were 0.10 ± 0.13 and 0.03 ± 0.07, respectively, for group B and 0.17 ± 0.14 and 0.08 ± 0.11, respectively, for group A.

Compared with conventional “epi-off” CXL, the corneal epithelium is removed by applying an infiltration enhancer that assists the thorough infiltration of riboflavin into the corneal stroma. The latter method can effectively eliminate the consequent adverse effects caused by the absence of an epithelial layer, such as corneal dehydration, subepithelial haze, and endothelial disruption [20]. Multiple researchers have proposed that “epi-on” CXL in fact, showed better outcomes for keratoconus patients and provided even more beneficial impact in clinical practice [21–24]. However, patients still experience different levels of stimulative ocular symptoms after transepithelial CXL because of riboflavin infiltration and UV light irradiation disruption. The major symptoms reported by the patients included foreign body sensation, dry eyes, photophobia, and various ranges of pain. This study demonstrated that the lens subsequently alleviated early postoperative symptoms, improving the patient’s ocular area. The scores of objective and subjective ocular symptoms for both groups experienced a clear decline after postoperative day 3 and then further decreased gradually from day 5 to day 7. Ozcan et al. [9] observed the effects of two types of SiH contact lenses that have been utilized in conventional CXL and found that the material and features of the bandage contact lens used after CXL facilitated corneal re-epithelialization. Acuvue Oasys, equipped with high oxygen permeability, are composed of a silicone hydrogel with a strong physical barrier. The immediate use of lenses after transepithelial CXL could effectively secure the exposed nerve ending and corneal epithelium and reduce the friction damage caused by blinking. Postoperative ocular signs reflected healing and the results of trans-CXL. Contact lenses with 8.8 mm base curve (BC) demonstrated a fine range of motion either in situ or upward gazing eye position, as well as sufficient accommodation at the central location, indicating that the lens is suitable for use in patients after rapid transepithelial CXL surgery.

In this study, we found that dry eye symptoms lasted the longest among all negative ocular signs. Several studies have demonstrated the correlation between CXL and dry eyes syndrome. In postoperative transepithelial CXL, a marked reduction has been observed in non-invasive tear break-up time (NITBUT), increased ocular surface disease index (OSDI), and impaired meibomian gland dysfunction [25, 26]. Furthermore, CXL can lead to corneal dehydration immediately after the procedure [27]. Hence, both “epi-on” and “epi-off” CXL procedures lead to dry eye or pseudo-dry eye features to a certain extent and CXL-caused dry eye symptoms shall be further investigated for corresponding postoperative intervention as several studies have indicated that symptoms alleviated within 6 months postoperatively in the majority of cases [28]. Yet the symptoms themselves are disturbing, even in the short-term, such as how the participants in the present research proposed that “dry eyes” was the longest distress amongst the examined subjective ocular sign.

This study has several limitations. As the present study was based on the subjective sensations of the participants, each patient’s identity and profile, such as educational background or comprehension ability, could equally influence the results. Further investigations that incorporate more subjective parameters are required. Moreover, tolerance of the lens for these two groups was not examined prior to the intervention, which may have also affected the results of the study.

In conclusion, 3 days is regarded as a desirable period for wearing corneal lenses in postoperative trans-epithelial CXL, showing satisfactory results in clinical observation, delivering better ocular sensation, and sufficient visual quality for patients with keratoconus.

## Data Availability

The data used in this study are available from the corresponding author upon request.

## Abbreviations

CXL: Corneal crosslinking
SiH: Silicon hydrogel
FDA: Food and Drug Administration
HPMC: Hydroxypropyl methylcellulose
VAS: Visual analogue scale
ANOVA: Analysis of variance
rANOVA: Repeated measures analysis of variance
BC: Base curve
NITBUT: Non-invasive tear break-up time
OSDI: Ocular surface disease index

## References

[1] D'Oria F et al. Refractive surgical correction and treatment of keratoconus. Surv Ophthalmol. 2023. 10.1016/j.survophthal.2023.09.005 10.1016/j.survophthal.2023.09.005 37774800

[2] Gordon-Shaag A, Millodot M, Shneor E, Liu Y (2015). The genetic and environmental factors for keratoconus. Biomed Res Int.

[3] Santodomingo-Rubido J (2022). Keratoconus: an updated review. Cont Lens Anterior Eye.

[4] Wu D (2021). Corneal cross-linking: the evolution of treatment for corneal diseases. Front Pharmacol.

[5] Hafezi F (2022). Corneal cross-linking: epi-on. Cornea.

[6] Agarwal R, Jain P, Arora R (2022). Complications of corneal collagen cross-linking. Indian J Ophthalmol.

[7] Jacob S (2014). Contact lens-assisted collagen cross-linking (CACXL): a new technique for cross-linking thin corneas. J Refract Surg.

[8] Plaka A (2013). Efficacy of two silicone-hydrogel contact lenses for bandage use after photorefractive keratectomy. Cont Lens Anterior Eye.

[9] OzarslanOzcan D, Ozcan SC (2021). Efficacy of two silicone-hydrogel bandage contact lenses after corneal crosslinking. Clin Exp Optom.

[10] Severinsky B, Wajnsztajn D, Frucht-Pery J (2013). Silicone hydrogel mini-scleral contact lenses in early stage after corneal collagen cross-linking for keratoconus: a retrospective case series. Clin Exp Optom.

[11] Uysal BS, Ozmen MC, Yuksel M, Aydın B, Bilgihan K (2023). Comparison of safety and efficacy of silicone hydrogel contact Lens-assisted CXL and accelerated CXL in keratoconus patients with thin corneas. Eur J Ophthalmol.

[12] Chalmers RL, Gleason W (2013). Overview of contact lens postmarket surveillance in the United States: system and recent study results. Eye Contact Lens.

[13] Zhang X (2018). Conventional and transepithelial corneal cross-linking for patients with keratoconus. PLoS ONE.

[14] Hawker GA, Mian S, Kendzerska T, French M (2011). Measures of adult pain: Visual Analog Scale for Pain (VAS Pain), Numeric Rating Scale for Pain (NRS Pain), McGill Pain Questionnaire (MPQ), Short-Form McGill Pain Questionnaire (SF-MPQ), Chronic Pain Grade Scale (CPGS), Short Form-36 Bodily Pain Scale (SF-36 BPS), and Measure of Intermittent and Constant Osteoarthritis Pain (ICOAP). Arthritis Care Res (Hoboken).

[15] Kocluk Y, Cetinkaya S, Sukgen EA, Günay M, Mete A (2017). Comparing the effects of two different contact lenses on corneal re-epithelialization after corneal collagen cross-linking. Pak J Med Sci.

[16] van der Valk Bouman ES (2021). Pain mechanisms and management in corneal cross-linking: a review. BMJ Open Ophthalmol.

[17] Mazzotta C (2008). Corneal healing after riboflavin ultraviolet-A collagen cross-linking determined by confocal laser scanning microscopy in vivo: early and late modifications. Am J Ophthalmol.

[18] Shetty R (2014). Profile of microbial keratitis after corneal collagen cross-linking. Biomed Res Int.

[19] Shetty R (2023). Cold bandage contact lens use reduces post-photorefractive keratectomy or corneal collagen-crosslinking pain perception in patients. Indian J Ophthalmol.

[20] D’Oria F, Palazón A, Alio JL (2021). Corneal collagen cross-linking epithelium-on vs. epithelium-off: a systematic review and meta-analysis. Eye Vis (Lond).

[21] Cifariello F (2018). Epi-off versus epi-on corneal collagen cross-linking in keratoconus patients: a comparative study through 2-year follow-up. J Ophthalmol.

[22] Akram S, Momin S, Malik B, Sirang Z (2022). Outcomes of epi-on collagen cross-linkage procedure assessed in progressive keratoconus patients. Cureus.

[23] Hill J (2020). Optimization of oxygen dynamics, UV-A delivery, and drug formulation for accelerated epi-on corneal crosslinking. Curr Eye Res.

[24] Ng SM, Ren M, Lindsley KB, Hawkins BS, Kuo IC (2021). Transepithelial versus epithelium-off corneal crosslinking for progressive keratoconus. Cochrane Database Syst Rev.

[25] Akgöz H, Fındık H, Aslan MG (2022) Evaluation of tear parameters and meibomian gland morphology in keratoconus patients after epithelial-on corneal cross-linking. Eur J Ophthalmol, 11206721221118740. 10.1177/11206721221118740 10.1177/11206721221118740 35929885

[26] Balıkçı AT, Ulutaş HG (2023). Evaluation of corneal parameters and Meibomian gland alterations after corneal cross-linking in patients with progressive keratoconus. Eye Contact Lens.

[27] Kontadakis GA (2013). In vitro effect of corneal collagen cross-linking on corneal hydration properties and stiffness. Graefes Arch Clin Exp Ophthalmol.

[28] Taneri S, Oehler S, Asimellis G, Kanellopoulos AJ (2013). Influence of corneal cross-linking for keratoconus on several objective parameters of dry eye. J Refract Surg.

